# The priority of goal-relevant information and evolutionarily threatening information in early attention processing:Evidence from behavioral and ERP study

**DOI:** 10.1038/s41598-020-65062-5

**Published:** 2020-05-14

**Authors:** Yuting Liu, Pei Wang, Guan Wang

**Affiliations:** 1 Department of Psychology, Shanghai Normal University, Shanghai, 200234 China; 2 Faculty of Education, East China Normal University, Shanghai, 200062 China; 3 School of Education Science, Huaiyin Normal University, Huaian, 223001 China

**Keywords:** Attention, Attention, Aggression, Aggression

## Abstract

Previous studies have demonstrated that evolutionarily threatening information and goal-relevant information can both capture attention. However, some studies have suggested that goal-relevant information is prioritized over evolutionarily threatening information, while some studies have shown the opposite conclusion. The aim of the present study was to investigate the attention advantage by presenting evolutionarily threatening information and goal-relevant information simultaneously. Three conditions were presented in this study: evolutionarily threatening information + an irrelevant stimulus, goal-relevant information + an irrelevant stimulus, and evolutionarily threatening information + goal-relevant information. The behavioral results showed no attentional bias toward evolutionarily threatening information in the two conditions including evolutionarily threatening information; in the two conditions including goal-relevant information, participants showed attentional bias toward goal-relevant information in both. However, the ERP results showed that in the two conditions including evolutionarily threatening information, a significantly stronger N2pc response was seen for evolutionarily threatening information than for the other types of pictures, and goal-relevant information produced a significantly stronger N2pc response than that for an irrelevant stimulus. The abovementioned results indicated that in the earlier stage of attention, both evolutionarily threatening information and goal-relevant information have attention processing advantages over irrelevant stimuli; furthermore, attention was captured by evolutionarily threatening information faster than it was by goal-relevant information.

## Introduction

Focusing limited attentional resources on potentially dangerous information can substantially increase an individual’s chances of survival. For example, when a threatening snake and a nonthreatening butterfly appear at the same time, the individual automatically prioritizes threatening information to respond appropriately^1,2^. In particular, a wealth of studies have shown that threatening information attracts attention. Various kinds of threatening stimuli, such as angry faces^3,4^, dangerous animals^5–7^, and violent situations^8^, have been included in these studies. Recent evidence has revealed that the automatic processing of threatening information is inherent. Specifically, evolutionarily threatening information such as snakes draw more automatic attention than other threatening information^9^. According to the snake detection hypothesis, snakes can pose deadly threat to human during the process of human evolution, and as a result, the detection of snakes hardly depends conscious cognition^10^. Not only do adults turn their attention toward evolutionarily threatening faster^11,12^ but infants do so, as well^13–15^. The automatic processing of evolutionarily threatening information can be explained by the direct pathway theory, which suggests that visually evolutionarily threatening information is processed automatically through specific subcortical pathways: evolutionarily threatening information is transmitted directly through the retina to the superior colliculus of the thalamus and then through the pulvinar nucleus of the thalamus to the amygdala. Thus, evolutionarily threatening information is processed automatically and, thus, faster than nonthreatening information^16^.

A few studies have shown that goal-relevant information can also capture attention^17–19^. Goal-relevant information includes information about a goal that an individual currently aims to achieve^20^. For example, Vogt and colleagues found that when participants were informed that they would score points for responding to the target words (“sheep” and “field”)‚ these words automatically captured attention^21^. Even when participants completed a task that required more cognitive resources, goal-relevant information presented as an interfering stimulus still captured attention^22^. Moreover, goal-relevant information was prioritized by attentional resources when it was presented subliminally^23^. These findings can be explained by the biased competition model, which states that attention focuses on information relevant to the individual’s current goals while ignoring target irrelevant information^24–26^. Phillips, Kambi and Saalmann^27^ further found the neural pathway that prioritized the processing of goal-relevant information: within the prefrontal-thalamus pathway, goal-driven attention processing occurs at the earliest stage of sensory processing.

Thus, both evolutionarily threatening information and goal-relevant information capture attention automatically. However, when evolutionarily threatening information and goal-relevant information are presented at the same time, which type of stimuli will be prioritized in the competition for attentional resources? We often encounter such situations in our daily life. For instance, while we are dealing with urgent work, a spider may suddenly come into our vision. Will the sight of a spider, as threatening information, quickly capture our attention and even interrupt the task we are performing? Some researchers have considered that the capturing of attention by evolutionarily threatening information occurs via bottom-up processing and that the capturing of attention by goal-relevant information occurs via top-down processing^26,28^. According to Theeuwes^29^, early attentional selection is only determined by the bottom-up selection mechanism and is not affected by the observer’s strategy. In other words, when threatening information and goal-relevant information are presented at the same time, evolutionarily threatening information will capture attention first, and this capture will not be affected by the goal-relevant information. This view is partially supported by a study showing that evolutionarily threatening information dominates during the facilitated attention stage^30^. However, Vogt, De Houwer, Crombez and Van Damme^20^ preliminarily explored the competition between threatening information and goal-relevant information for attentional resources by using a dot-probe task and target task and found that goal-relevant information captured attention faster than evolutionarily threatening information. Therefore, whether evolutionarily threatening information or goal-relevant information will capture attention first when presented simultaneously is still controversial.

The inconsistent results in the abovementioned studies may be caused by the different attention stages that were examined in each experiment: the stimuli in the first study were presented for 47 ms^30^, in the early stage of attention, while the stimuli in the other study were presented for 350 ms^20^, in the late stage of attention. In our study, we used ERPs to further explore the essence of the competitive advantage of threatening information and goal-relevant information on attentional resources in the early stage of attention. Notably, N2pc is an ERP component closely related to spatial selective attention^31^, consisting of a negative wave that occurs in the posterior lobe contralateral to the target stimulus at approximately 200 ms^32^. The N2pc component is an indicator of the advantages of a stimulus in early attentional processing. In a number of studies, N2pc has shown that threatening information usually captures attention faster than irrelevant stimuli^33–36^. Similarly, N2pc has shown that goal-relevant information can also capture attention with a higher priority than neutral stimuli^31,37,38^. According to previous research^39–41^, the mean amplitude values were measured at lateral posterior electrodes P7 and P8 to compute N2pc.

In the present study, we used ERPs in combination with behavioral measures in the dot-probe task to explore the attentional competitive advantage between goal-relevant information and threatening information in the earlier stage of attention. To manipulate goal-relevant information, we established a goal task in which participants were asked to respond to one specific kind of picture. We included three conditions in the dot-probe task: evolutionarily threatening information + an irrelevant stimulus, goal-relevant information + an irrelevant stimulus, and evolutionarily threatening information + goal-relevant information. Based on the views above, we predict that in the early stage of attention, the N2pc component elicited by threatening information and goal-relevant information will be stronger than that elicited by irrelevant stimuli; moreover, evolutionarily threatening information will trigger a stronger N2pc than that triggered by goal-relevant information.

## Results

### Behavioral results

The data (correct response time) were analyzed with repeated measures analysis of variance (ANOVA) using picture pairs (evolutionarily threatening information + irrelevant stimulus, goal-relevant information + irrelevant stimulus, evolutionarily threatening information + goal-relevant information) and dot and stimulus location (congruent, incongruent) as within-subjects factors. The interaction between the factors was significant, *F*(2, 17) = 28.19, *p* < 0.001, η^2^_p_ = 0.61. Simple effects analysis found that in evolutionarily threatening information + irrelevant stimulus picture pairs, there was no significant difference in response times between congruent evolutionarily threatening information and incongruent evolutionarily threatening information. In goal-relevant information + irrelevant stimulus picture pairs, the response time for trials with goal-relevant congruence (432.43 ms) was significantly lower than that for trials with goal-relevant incongruence (480.27 ms), *F*(1, 18) = 56.47, *p* < 0.001, η^2^_p_ = 0.76. In evolutionarily threatening information + goal-relevant information picture pairs, the response time on trials with evolutionarily threatening information congruence (470.24 ms) was significantly higher than that for trials with evolutionarily threatening information incongruence (427.81 ms), *F* (1, 18) = 17.41, *p* = 0.001, η^2^_p_ = 0.49 (See Table 1).

**Table 1:** Behavioral performance (Mean ± SD).

Picture pairs	Congruence		Incongruence		Difference value	
	*M*	*SD*	*M*	*SD*	*M*	*SD*
evolutionarily threatening information + irrelevant stimulus	474.83	20.14	482.96	21.95	−8.14	7.08
goal-relevant information + irrelevant stimulus	432.43	15.91	480.27	16.99	−47.84^*^	6.37
evolutionarily threatening information + goal-relevant information	470.24	18.69	427.81	11.78	42.43^*^	10.17

### ERP results

**Figure 1:**
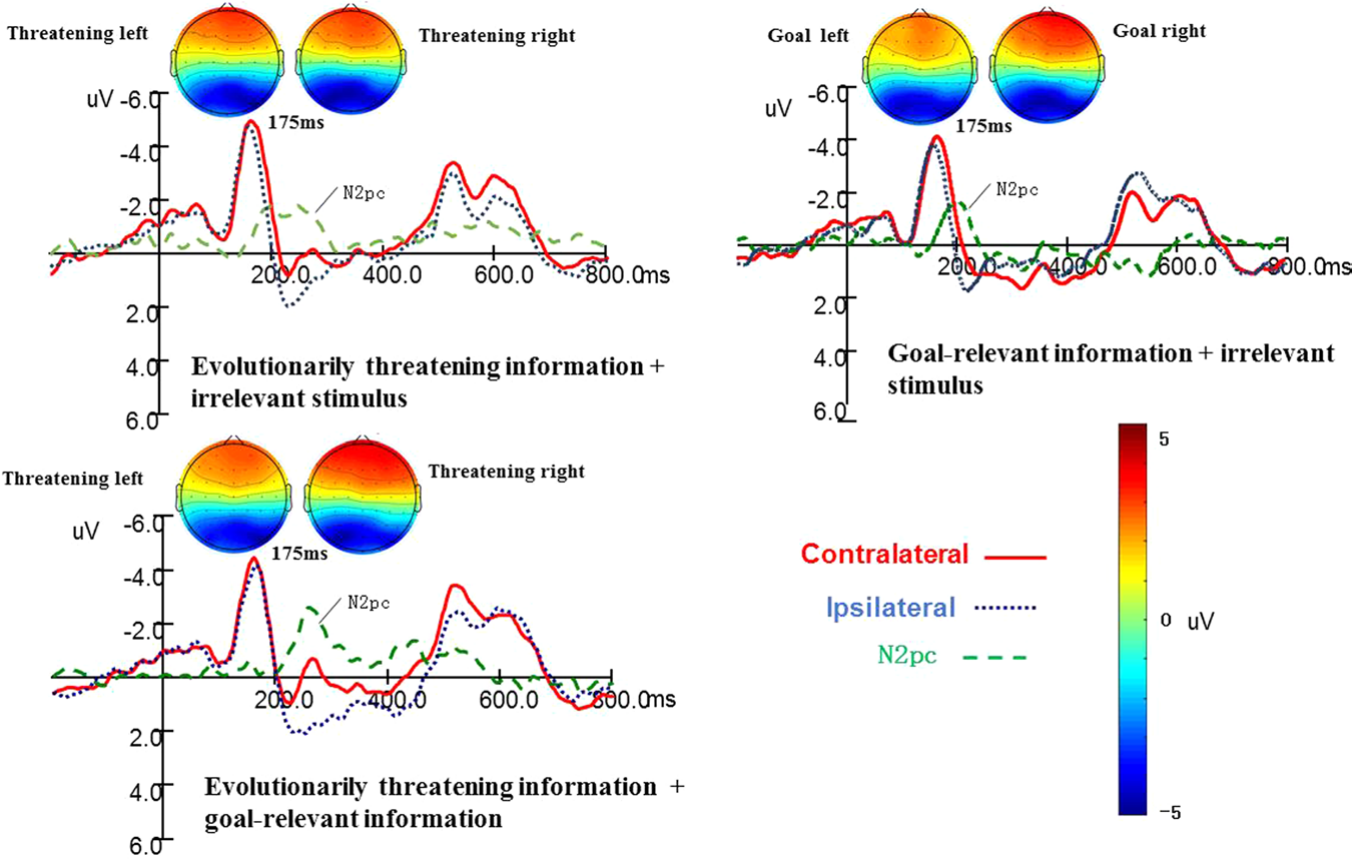
Grand mean ERPs of N2pc in contralateral and ipsilateral conditions recorded at P7 and P8 and the differences between contralateral and ipsilateral conditions. In the evolutionarily threatening information + irrelevant stimulus and evolutionarily threatening information + goal-relevant information trials, contralateral signifies waveforms contralateral to the threatening image; In the goal-relevant information + irrelevant stimulus trials, contralateral signifies waveforms contralateral to the goal-relevant information. Topographical voltage maps in each condition were presented and the latency was 175 ms. In the evolutionarily threatening information + irrelevant stimulus, the left map showed the results of trials that evolutionarily threatening information appeared on the left screen, while the right map showed the results of trials that evolutionarily threatening information appeared on the right screen.

In evolutionarily threatening information + irrelevant stimulus and evolutionarily threatening information + goal-relevant information trials, N2pc was measured by the difference wave as calculated by the waveform contralateral to the threatening image minus the waveform ipsilateral to the threatening image. In goal-relevant information + irrelevant stimulus trials, N2pc was measured by the difference wave as calculated by the waveform contralateral to the threatening image minus the waveform ipsilateral to the goal-relevant information. In the three conditions, a paired sample t-test was used to compare the difference between the contralateral wave and ipsilateral wave. In evolutionarily threatening information + irrelevant stimulus trials, the difference between contralateral and ipsilateral waves was significant, *t*(18) = 4.15, *p* < 0.001, *d* = 0.95. The amplitude contralateral to the threatening image (−1.57 ± 1.07 μV) was significantly higher than that ipsilateral to the threatening image (−0.17 ± 1.51 μV). The N2pc contralateral-minus-ipsilateral value was *M* = −1.40 uV, *SD* = 1.47. In goal-relevant information + irrelevant stimulus trials, the difference between contralateral and ipsilateral waves was significant, *t*(18) = 3.15, *p* = 0.001, *d* = 0.88. The amplitude contralateral to the goal-relevant information (−1.29 ± 1.25 μV) was significantly higher than that ipsilateral to goal-relevant information (−0.58 ± 1.25 μV). The N2pc contralateral -minus- ipsilateral value was *M* = −0.71 uV, *SD* = 0.82. In evolutionarily threatening information + goal-relevant information trials, the difference between the contralateral and ipsilateral waves was significant, *t*(18) = 3.79, *p* = 0.001, *d* = 0.88. The amplitude contralateral to the threatening image (−1.27 ± 2.08 μV) was significantly higher than that ipsilateral to the threatening image (−0.21 ± 2.18 μV). The N2pc contralateral-minus-ipsilateral value was *M* = 1.06 uV, *SD* = 1.22 (See Fig. 1).

## Discussion

The present study, for the first time, investigated the attention processing advantages that evolutionarily threatening information and goal-relevant information receive using ERPs. The ERP results showed that evolutionarily threatening information and goal-relevant information captured attention faster than irrelevant stimuli. Moreover, evolutionarily threatening information captured attention faster than goal-relevant information.

The behavioral data showed that there was no attentional bias toward evolutionarily threatening information versus either irrelevant stimuli or goal-relevant information, indicating that there is no advantage of attention capture for evolutionarily threatening information in competition with other stimuli; in both goal-relevant information + irrelevant stimulus picture pairs and evolutionarily threatening information + goal-relevant information picture pairs, participants showed attentional bias toward goal-relevant information. These results were consistent with the results of previous research that used the dot-probe paradigm to compare the prioritization of goal-relevant information and threatening information in attention processing^20^. Here, we explained why there was no attentional bias toward threatening information from theoretical and technical perspectives. In theory, there are two reasons. On the one hand, the behavioral data showed that participants avoided evolutionarily threatening information in the later stage of attention. Previous studies have shown that in the early stage of attention, individuals will focus their attention on evolutionarily threatening information^42^. In the later stages, individuals tend to avoid threatening information^43,44^. On the other hand, due to the rewards in goal-relevant task, participants shifted their attention to the goal-relevant task later^26,45^. Considering experimental manipulation, the behavioral indicator of dot-probe task is an unreliable measure of attention allocation in nonclinical samples^46,47^.

Although we found no evidence of attentional bias toward evolutionarily threatening information using reaction times, ERPs revealed that there was an attentional bias toward both evolutionarily threatening information and goal-relevant information, as reflected by N2pc. In evolutionarily threatening information + irrelevant stimulus trials and goal-relevant information + irrelevant stimulus trials, both evolutionarily threatening information and goal-relevant information triggered a larger N2pc amplitude than irrelevant stimuli. These results are consistent with our expectations and have been confirmed by a previous study. Kappenman and his colleagues^48^ used the dot-probe paradigm to verify that when threatening information and an irrelevant stimulus were presented at the same time, participants would prioritize the threatening information in their attention, and the contralateral brain region would generate larger amplitude of N2pc in response to the threatening information. Similarly, researchers found that goal-relevant information also caused a higher N2pc amplitude^49,50^. These phenomena can be explained by the biased competition model^24^, lateral striatal neurons can only represent one object at a time, and when multiple objects appear in the field of vision at the same time, these objects will compete for the resources of this neuron, resulting in the processing of only one object. Therefore, when multiple objects appear in the visual field, the most salient stimulus (evolutionarily threatening information or goal-related stimulus) will take priority over an irrelevant stimulus to capture attention^51^.

More importantly, the ERP results of this study first found that when evolutionarily threatening information and goal-relevant information are presented at the same time, the evolutionarily threatening information will capture attention with priority. In other words, in the early stage of attention, evolutionarily threatening information captured attention faster than temporarily goal-relevant information. Previous research suggested evolutionarily threatening information can lead to the “bottom-up” capture of attention^52–54^. This result supported Theeuwes’ opinion^29^ that when people first pay attention to multiple objects, visual selection is dominated by bottom-up stimuli (evolutionarily threatening information) due to the specific neural pathway processing of evolutionarily threatening information^15^; the stronger the attentional bias is toward the evolutionarily threatening information, the stronger the positive connection between the amygdala and the anterior cingulate gyrus is^55,56^. In addition, this result is of great significance to the survival of humans. Since evolutionarily threatening information can pose deadly threat to human during the process of human evolution^57^, people place more emphasis on safety than goals that would bring value. Prevailing accounts of attention to threat^58–61^ consider that threatening information always takes priority because rapidly reacting to this type of information could guarantee survival.

Actually, our study is not the first to attempt to understand the competition between bottom-up stimuli and top-down stimuli. However, previous studies have failed to find an appropriate experimental task to explore this question. For example, Mounts, McCarley and Terech^62^ adopted a target-decoy paradigm to study the competition between bottom-up stimuli and top-down stimuli. The target (top-down) and decoy (bottom-up) stimuli were differently colored singleton letters among an array of gray distractors. In this paradigm, both the target and decoy stimuli were initially processed due to their bottom-up salience because they were both colored objects. There was no strict distinction between top-down and bottom-up processing in this paradigm. Hilimire and Corballis^28^ found that bottom-up stimuli did not elicit N2pc using a target-decoy paradigm. This conclusion is unreliable because of the inappropriate experimental paradigm. In the present study, we completely distinguish top-down and bottom-up stimuli, which were represented by goal-relevant information and evolutionarily threatening information. Therefore, we drew different conclusions, showing that bottom-up stimuli (threatening information) capture attention faster.

The limitations of the present study are discussed as follows. First, we chose snake images as the threatening information, as many studies have shown that people are born afraid of snakes^61,63^; however, this stimulus is not common in daily life. To improve the ecological validity of the study, we could choose socially threatening information, such as angry faces, for future studies. Second, we explored attention processing characteristics in the early stage of attention using ERPs. According to Theeuwes’ opinion^29^, threatening information captures attention faster than goal-relevant information in the early stage, but later, goal-relevant information will take attentional priority. Thus, we could focus on later ERP components, which can reflect attentional selection in later stages, in future research.

In summary, both evolutionarily threatening and goal-relevant information took priority over irrelevant stimuli to capture attention. Importantly, when evolutionarily threatening information and goal-relevant information were presented at the same time, the N2pc component in the early stage of attention showed that evolutionarily threatening information captured attention first. This result may be caused by particularities of the neural pathway that processes evolutionarily threatening information and the bottom-up processing of stimuli.

## Material and Methods

### Participants

Twenty-one college students (9 females and 12 males) with a mean age of 23.2 years (*SD* = 1.25) participated in this study. Participants were recruited from Shanghai Normal University and signed informed consent forms before participating. This study was approved by the local ethics committee of Shanghai Normal University and was conducted in accordance with the Declaration of Helsinki (2013). All participants were native Chinese speakers with no reported neurological disorders. Each participant received a certain reward amount after the experiment. We calculated the sample size before the experiment using G-power, we set the effect size d value as 0.80, the α value as 0.05, and the power value as 0.80; the sample size was 15. As a convention proposed for general use, d = 0.80 indicates a large effect size, and the power is 0.80^64^.

### Design and materials

**Figure 2:**
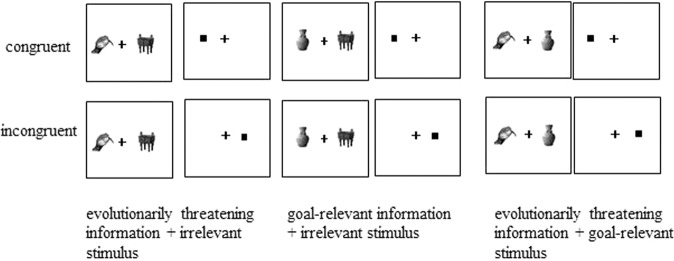
Three types of picture pairs appeared in the dot-probe task.

This study was conducted by adopting a within-subject design, with 3 (picture pairs: threatening information + irrelevant stimulus, goal-relevant information + irrelevant stimulus, threatening information + goal-relevant information) × 2 (dot and stimulus location: congruent, incongruent) factors (See Fig. 2). For the different picture pairs, the congruent condition means different things. In threatening information + irrelevant stimulus picture pairs, when the dot and the threatening information appeared at the same location, they were congruent; in goal-relevant information + irrelevant stimulus picture pairs, when the dot and the goal-relevant information appeared at the same location, they were congruent; in threatening information + goal-relevant information picture pairs, when the dot and threatening information appeared at the same location, they were congruent.

Four pictures displayed snakes as the threatening information (142, 146, 149, and 152), four pictures displayed vase as the goal-relevant information (849, 465, 836, and 329) and four pictures displayed a desk as the irrelevant stimulus (352, 477, 777, and 315); all stimuli were selected from the CAPS (Chinese Affective Picture System)^65^. The original picture backgrounds were replaced with a white background, and the pictures were transformed to be equal in size and have the same average gray values. We used three types of color blocks (green, gray and blue) as filler stimuli in the goal task. Finally, we selected four neutral pictures from the CAPS for the practice phase.

Forty-four undergraduates (22 females) who did not participate in the main experiment were selected to rate how threatening the materials were on a scale from 1 (not threatening at all) to 7 (extremely threatening). Variance analysis was conducted on the ratings of the three types of materials. There were significant differences in the level of threat between the threatening information (*M* = 1.93, *SD* = 0.93), goal-relevant information (*M* = 1.78, *SD* = 0.96) and the irrelevant stimuli (*M* = 5.93, *SD* = 1.25). *F* (2, 129) = 218.32, *p* < 0.001, η^2^_p_ = 0.11. Specifically, the level of threat for the threatening information was significantly higher than that of the goal-relevant information (*p* < 0.001) or the irrelevant stimuli (*p* < 0.001). There was no significant difference in the level of threat between the goal-relevant information and the irrelevant stimuli (*p* = 0.512).

A pilot study was conducted before the main experiment to verify whether there was interference caused by the shape and concept of the stimuli and to test the effectiveness of target task manipulation. Thirty college students (17 females and 13 males) with a mean age of 23.03 years (*SD* = 1.52) from Shanghai Normal University participated in the pilot study. The procedure was almost the same with the main experiment, and the only difference was that participants were informed to respond to both “snake” and “vase” in the goal task. In other words,“snake”and“vase”were both goal-relevant information. The correct reaction time of dot probe task was analyzed with repeated measures analysis of variance (ANOVA) using picture pairs (snake + desk, vase + desk, snake + desk) and dot and stimulus location (congruent, incongruent) as within-subjects factors. The interaction between the factors was significant, *F* (2, 28) = 5.58, *p* = 0.009, η^2^_p_ = 0.29. Simple effects analysis found that in snake + desk picture pairs, the response time for trials with snake congruence (501.09 ms) was significantly lower than that for trials incongruence (527.99 ms), *F* (1, 29) = 13.29, *p* = 0.001, η^2^_p_ = 0.31. In vase + desk picture pairs, the response time for trials with vase congruence (493.63 ms) was significantly lower than that for trials incongruence (537.57 ms), *F* (1, 29) = 27.51, *p* < 0.001, η^2^_p_ = 0.49. In snake + vase picture pairs, there was no significant difference between the response time for trials with snake congruence and that for trials with snake incongruence. The results showed that the manipulation of target stimuli (snake and vase) was successful, and the concept and shape of the materials would not interfere with the results.

### Procedures

Participants were seated approximately 60 cm from the monitor. The participants were asked to perform two tasks per session. In the dot-probe task, at first, a fixation cross (5 mm) was presented in the middle of the screen for 500 ms, along with picture pairs (two pictures, with one presented on the left side of the screen and the other presented on the right) appeared on the screen for 350 ms; each picture was 4.6 cm tall and 6.1 cm wide, and the distance between the center of each picture and the fixation cross was 4.6 cm. Immediately after the picture pairs disappeared, a black dot (0.5 × 0.5 cm) appeared on the left or right side of the screen at the location where the picture had been displayed. If the black dot appeared on the left, participants were instructed to press “O” with their right index finger to react; if the black dot appeared on the right, participants were asked to press “P” with their right middle finger to react. The offset of the black dots occurred after response or after 1500 ms.

**Figure 3:**
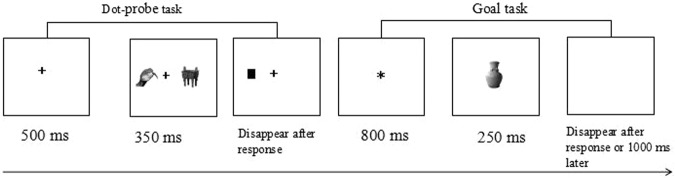
Flow chart of one trial. A fixation cross was presented in the middle of the screen for 500 ms, along with one of the picture pairs, appearing on the left or the right side of the screen, for 350 ms. After the offset of the picture pair, a black dot would appear on the left or right side of the screen. If the black dot appeared on the left, participants were instructed to press the “O” key with their right index finger to react; if the black dot appeared on the right, participants were instructed to press “P” with their right middle finger to react. The offset of the black dot occurred after a response or after 1500 ms. After that, an “*” appeared in the middle of the screen for 800 ms; then, a picture appeared in the middle of the screen for 250 ms, followed by a blank screen. If the picture was goal-relevant, participants were instructed to press the spacebar with the left hand; otherwise, they were told not to respond.

The goal task started 600 ms after the dot-probe task finished. The goal task started with the appearance of an “*” in the middle of the screen for 800 ms; then, a picture appeared in the middle of the screen for 250 ms, after which a blank screen appeared. If the picture was goal-relevant, participants were instructed to press the spacebar with the left hand; otherwise, they were instructed not to respond. Correct reactions to the goal-relevant picture were followed by a feedback screen indicating that the reaction was correct. Incorrect reactions or no reaction were followed by error feedback. The feedback picture was presented for 500 ms. The task ended when the participant responded or the blank screen had been presented for 1000 ms. (see Fig. 3)

Practice phase: In the dot-probe task, participants were asked to focus a**t**tention on the “+” and to respond to the dot location as quickly and accurately as possible. In the goal task, participants were informed that a picture would be presented in the middle of the screen after they responded to the dot. If this picture was a cap, they were instructed to press the spacebar key with the left hand. Participants completed 16 practice trials, until their accuracy was higher than 0.8, and then, they began the test phase.

Test phase: First, we showed the participants pictures of vase and told them that they would be asked to detect these pictures in the goal task. Participants would win 1 point if they correctly responded to the goal-relevant picture during the goal task. In the test phase, each picture pair (evolutionarily threatening information + irrelevant stimulus, goal-relevant information + irrelevant stimulus, and evolutionarily threatening information + goal-relevant information) appeared in 120 trials, resulting in 360 trials in total. Each type of picture was presented on the left in half of the trials and on the right in the other half. For each picture pair, the dot was presented at the location where one category had been presented in half of the trials. In the goal task phase, each type of picture (evolutionarily threatening information, goal-relevant information, irrelevant stimulus or filler stimulus) appeared in 90 trials. The dot-probe task and goal tasks alternated with an intertrial interval of 600 ms. The order of the presentation of the stimuli in both tasks was random for each participant. Notably, the picture pairs that were presented in the dot-probe task could not predict the picture that would appear in the subsequent trial of the goal task.

### Electrophysiological recordings and data analysis

EEG collection and analysis were performed using NeuroScan4.4, and signals were acquired using 64 Ag/AgCl electrodes attached to an elastic cap. The left mastoid was used as the reference electrode for online data recording, and electrodes recording horizontal electrooculograms (EOG) were placed 10 mm from the lateral corner of each eye. Vertical EOG was measured via electrodes placed 10 mm above and below the left eye. EEGs were sampled at 500 Hz by NeuroScan Synamps2 amplifiers using a 0.01-Hz to 100-Hz sampling rate. The impedance between the scalp and the electrodes was less than 5 kΩ. The data of two participants were deleted because of the presence of too many electrical artifacts; thus, 19 participants were included in the statistical analysis. We rejected trials that were contaminated by blinks, eye movements or excessive muscle activity (voltage exceeded ± 80 in any channel). High frequency noise was further low-pass filtered offline at 30 Hz. We primarily analyzed ERPs elicited by the picture pairs, and epochs were defined as the change in ERPs within 800 ms after the onset of the picture pairs and the baseline was the signal 200 ms prestimulus.

To determine whether attention was preferentially allocated to one of the picture pairs, we isolated the N2pc wave to the onset of the picture pairs at posterior electrode sites (P7 and P8)^40^. For example, for the evolutionarily threatening information + irrelevant stimulus pairs, we created separate waveforms for the hemisphere that was contralateral to the evolutionarily threatening information (i.e., left hemisphere electrode sites for threatening information presented on the right side and right hemisphere electrode sites for evolutionarily threatening information presented on the left side) and for the hemisphere that was ipsilateral to the threatening image (i.e., right hemisphere electrode sites for evolutionarily threatening information presented on the right side and left hemisphere electrode sites for evolutionarily threatening information presented on the left side). For goal-relevant information + irrelevant information pairs, the contralateral waveform signifies that in the hemisphere contralateral to the temporarily goal-relevant information, and the ipsilateral waveform signifies that in the hemisphere ipsilateral to the temporarily goal-relevant information. For the evolutionarily threatening information + goal-relevant information pairs, the contralateral waveform signifies that in the hemisphere contralateral to the evolutionarily threatening information, and the ipsilateral waveform signifies that in the hemisphere ipsilateral to the evolutionarily threatening information. We then created a contralateral-minus-ipsilateral difference waveform, and the N2pc component was calculated via the difference values of the contralateral and ipsilateral waves of each participant. The mean amplitude of the N2pc component was measured using an a priori time window of 150–250 ms following the onset of the picture pairs^48^. In the abovementioned three conditions, a paired sample t-test was used to compare the difference between the contralateral wave and ipsilateral wave respectively.

Mean reaction time and accuracy of the dot-probe task and goal task were recorded. The correct rate of all participants was higher than 0.92 in the dot-probe task, and the correct rate of all participants was higher than 0.94 in the goal task. The correct response time in the dot-probe task were analyzed with repeated measures analysis of variance (ANOVA) using picture pairs (evolutionarily threatening information + irrelevant stimulus, goal-relevant information + irrelevant stimulus, evolutionarily threatening information + goal-relevant information) and dot and stimulus location (congruent, incongruent) as within-subjects factors. In the dot-probe paradigm, if the response times in trials in which the evolutionarily threatening information and the dot were congruent were significantly lower than the response times in incongruent trials, which indicates that evolutionarily threatening information captures attention faster than the other stimuli^66^.
